# A Fog Computing-Based Device-Driven Mobility Management Scheme for 5G Networks

**DOI:** 10.3390/s20216017

**Published:** 2020-10-23

**Authors:** Sanjay Kumar Biswash, Dushantha Nalin K. Jayakody

**Affiliations:** 1 School of Computer Science and Robotics, National Research Tomsk Polytechnic University, 634050 Tomsk, Russia; skbismu@gmail.com; 2 Department of Computer Science and Engineering, NIIT University, Neemrana 301705, Rajasthan, India; 3 Centre for Telecommunications Research, School of Engineering, Sri Lanka Technical Campus, Padukka 10500, Sri Lanka

**Keywords:** fog networks, 5G networks, device-driven communications, data-centric networking

## Abstract

The fog computing-based device-driven network is a promising solution for high data rates in modern cellular networks. It is a unique framework to reduce the generated-data, data management overheads, network scalability challenges, and help us to provide a pervasive computation environment for real-time network applications, where the mobile data is easily available and accessible to nearby fog servers. It explores a new dimension of the next generation network called fog networks. Fog networks is a complementary part of the cloud network environment. The proposed network architecture is a part of the newly emerged paradigm that extends the network computing infrastructure within the device-driven 5G communication system. This work explores a new design of the fog computing framework to support device-driven communication to achieve better Quality of Service (QoS) and Quality of Experience (QoE). In particular, we focus on, how potential is the fog computing orchestration framework? How it can be customized to the next generation of cellular communication systems? Next, we propose a mobility management procedure for fog networks, considering the static and dynamic mobile nodes. We compare our results with the legacy of cellular networks and observed that the proposed work has the least energy consumption, delay, latency, signaling cost as compared to LTE/LTE-A networks.

## 1. Introduction

The ubiquity of mobile devices is one of the prime objectives in modern cellular networks. It expands the system computing, service management to multiple connected end-users, and supports many peripheral technologies such as cellular networks, IoT, Wi-Fi, and Bluetooth. The modern cellular network is an amalgamation of fifth-generation (5G) networks and other communication technologies. 5G mobile cellular network is a new design to support the higher data rates, dense network connectivity (millions of devices per square km), lower transmission latency, very high node mobility, the reliable and ubiquitous user interface to mobile nodes, but it has many major research challenges in real-time deployment [1]. The researcher found that 5G network expectations and ground reality have a big performance gap [2]. For example, the delay is 1000 times and latency is 100 times less than current network technologies [1]. In the sequel of 5G networks, fog-networks is proposed in Ref. [3,4,5]. They also propose a similar line of performance for ultra-dense deployment of mobile base stations (BSs) and frequent fog nodes installation to achieve QoS and QoE. It leads to higher transmission rates and very low latency to end-user devices.

Fog network follows the concept of cloud-backup at the remote level and front-end processing for computing devices and applications. The cloud-based service and network-enabled devices are interlinked with each other by fog nodes. They use an anonymous number of front-end users and internet on things (IoT) applications [6]. The pervasive nature of IoT-enabled mobile devices is helpful in daily-life activities. They provide utility, service-based applications, and network monitoring facilities. The proliferation of IoT devices has created a large network interface with sensors and actuators, which provide a delay-sensitive response to mobile users. The association of IoT devices, mobile nodes, and fog servers are coupled foregathers in fog networks. Fog network is the future of cellular communication system but also have many critical research challenges. It provides a massive number of end-users support, fast data access and processing, device-driven communication environment, and heterogeneous network interface [7,8]. These attractive key benefits play an important role to choose the fog networks as a future of telecommunication networks.

In this paper, we consider the long-term evolution (LTE) and LTE-Advance are the most recent, deployed cellular networks [9]. Therefore, we compare our results w.r.t it. LTE and LTE-A technologies use IP-based backbone network support. The home subscriber server (HSS) used to process the user’s and system-generated data, global MME-ID management is the part of this procedure [10]. It increases the processing time, communication delay, signaling overhead, and network registration cost [11,12]. These challenges motivate the researchers to produce a new network architecture such as fog networks.

The rest of the paper is organized as follows. Section 1 provides complete information about fog computing & cellular network, research challenges, motivations, and contributions of the paper. In Section 2, we discuss the future generation cellular network using fog computing, as it is one of the major contributions. Our next contribution is highlighted in Section 3, where we talk about device-driven communication procedures in the proposed network schema. In Section 4, we introduce a mobility management procedure for proposed network architecture. The advantage and open research challenges of the works are in Section 5. The performance analysis and comparison are available in Section 6. Section 7 is used for the conclusions and followed by listed references.

### 1.1. Fog Network-Integrated Cellular Communication System

Cloud computing technology support IoT and deployed in cellular networks, but it cannot satisfy the demand for fast multimedia communication and greedy users’ expectations with high node mobility [13]. Originally it was built to connect billions of smart-devices with IP-based backbone networks, to fill the hypothesis of “smart-heterogeneous network connectivity to mobile users” [14], but it was not an up-to-the-mark solution to improve the QoE. The proposed solution [14] has many challenges related to the massive number of node connections, robustness, and scalability, delay and latency, poor throughput, etc. Therefore researchers are looking forward to new alternative solutions. It was the core motivation for fog computing-based networking, where the end-users have easy access to computing services and applications [15].

To achieve better network performance, system-generated data should be sent over the cloud for further processing, especially, knowledge discovery and acquisition [16,17]. It will help the network and applications to identify the user’s credentials, network possibility, target node specification, and most reliable network route. As any mobile node sends the network registration request to associated BS/gNB/eNB, the BS forward it to the mobile switching center (MSC). The MSC needs the information (data) about the source and target mobile nodes. Thereafter, it may perform a network registration procedure to serve mobile nodes. This technique helps the system to take appropriate actions to connected devices for better network and user experience.

Fog networks are one of the most efficient modes of communication to fill the expectation of mobile users. It provides a new dimension for the next-generation cellular networks. It is a promising candidate to accommodate a high demand of mobile traffic with low latency [18,19]. Fog services expedite to computation, storage, and networking processes. Theses are hosted in the vicinity of end-users (edge of the network). Thus, it results in delay-sensitive mobile applications over the fastest-growing network-applications [20].

In Ref. [21], authors analyze the modern standardization for device-to-device (D2D) communication. This is helpful to effectively enable direct machine-to-machine (M2M) communication under the umbrella of 5G networks. However, the proposed work has a lack of cloud-based network services. In Ref. [22], the authors proposed a cloud radio access network (C-RAN) based framework by exploring the recent rapid development of cloud computing and cloud-network. They claimed suggested architecture is an ideal platform to support network function virtualization (NFV), software-defined networking (SDN), and artificial intelligence (AI) for 5G networks. The above-mentioned discussion about 5G and cutting-edge network technologies has several disadvantages such as network resource wastage, high delay, non-systematic storage, high energy consumption, and poor performance measurements [5]. The radio access networks (RAN), Internet multimedia services (IMS), and general packet radio service (GPRS) are the main attribute of 3G and beyond cellular networks. As cloud-based networking is introduced, the amalgamation of cloud and RAN became more complex. The fog network could be the best candidate to overcome these complexity i.e., RAN dependency, MoIP support with GPRS, etc. The fog networks paradigm is used to deal with the above-listed issues in next-generation cellular communication. It is a simple and effective design to introduce a new scheme to process the data, data management procedure, knowledge discovery, and other techniques to serve the edge of the connected device or associated network hardware. However, mostly edge devices have limited computational capabilities. Then, advanced computing services should be implemented in intermediate data centers/access points, and mobile devices [18,23].

### 1.2. Key Challenges in Fog Networks

The theoretical aspects of the fog networks are available in several pieces of literature, but real-life implementation missing to date [18,24]. To deploy the fog networks in real-time domain following research challenges must be addressed [25,26].

- **Dynamic node-mobility and mobility management:** The state-of-the-art for fog network opens several research challenges to deploy it for real-time dynamic environments, example are delay assisted by node mobility, data accessibility issue while mobile nodes are moving very fast, energy-efficient networks, mobility support for frequent moving users, etc. The listed issues are the most emerging areas of fog networks, where we should focus and try to implement high network mobility support for better network experience. As a mobile node changes the position from one gNB to another gNB, it requires an assistantship for efficient network registration and update procedure at the foreign position. To implement the fog network, the system requires frequent information retrieval and update over nearby fog data servers. This attribute sought the attention of researchers for better network mobility support.
- **Distributed communication protocol support:** As the mobile devices are proliferating with cloud computing. It is expected that there will be a huge demand for better QoE/QoS in the IoT enabled network-services area, it will be used by all mobile devices and network applications. The mobile nodes and cloud-based services should be merged with a software-based real-time network virtualization platform. The advances in network-based software cloud open a new era of mobile fog networks and computing, it moves towards the fog network operating system (fog OS). The fog OS help in computing, storage, and networking even closer to the end-users for better QoS/QoE [27]. It needs an open platform where the networks can work on the distributed environment without any barrier of network protocols and restrictions, as the IoT network prefers to do.
- **Data analytics and processing:** The gateway devices of fog networks are expected to have limited computing and processing capabilities. Thus, there is a requirement of module-based data analytical components and services. It will help the data to process at the end-user site (level), to establish the relation between the device and nearby fog data server [28]. It will support a large variety of IoT-enabled devices for smart applications, also capable to analyze the processed data at a local edge device level. Besides it, we can install-on-demand data analytic tools and applications, but this deployment needs a large database space and costly hardware at the end-user level. Most important point is to provide a necessary tool for all end-user devices, which can process generic real-time data sets [29].
- **Security and privacy:** As the connected devices are available over the network area and growing exponentially, the generated data, processed, and demanded data is also increase proportionally. The fog network is one of the possible techniques that can accommodate high volume data sets, therefore it opens the door to new challenges such as security and privacy of data. In the fog network data is very close to the edge devices then it needs more security and privacy than others [19].

### 1.3. Motivation and Contributions

The fog computing-based cellular network is not deployed in the telecommunication system, also research and challenges are not fully discussed in the literature, therefore fog networks real-time implementation is very limited to date. It motivates us to extend our research towards fog computing-based telecommunication networks. Thereafter, we propose the following cutting edge contributions within the domain of fog networks.

- The 5G is modern cellular communication technology, and fog computing have high-computing and service capabilities. We combine these two technologies and propose a fog network-based communication architecture for 5G and beyond (5GB) telecommunication systems, as discussed in Section 2.
- 5G network supports the device-centric communication procedure, it is an advancement of the D2D communication procedure. The device-centric communication needs a breakthrough for performance improvement and it’s deployment with telecommunication networks. In this paper, we extend our research in device-centric communication to a new level called device-driven communication. We propose a device-driven communication methodology for the 5GB system i.e., fog network, as discussed in Section 3.1. It has two variations of communication such as for static mobile users and for dynamic mobile users, they are discussed in Section 3.2 and Section 3.3, respectively.
- Mobility management is a fundamental feature in the cellular network. Therefore, we propose a mobility management procedure for fog computing-based device-driven networks in Section 4. As the mobile nodes change their position then the mobile-dataset changes in respective fog nodes, this process is dealt with mobility management as discussed in Section 4.1. Thereafter the call delivery is one of the most challenging tasks. The details of the proposed call delivery procedure are discussed in Section 4.2.
- The fog computing-based device-driven communication is a new dimension of mobile cellular networks. It has several advantages and cutting edge open research areas, as they are highlighted in Section 5. Finally, the performance analysis and detail result discussion is available in Section 6.

As per the best of the authors’ knowledge, the proposed work is completely different from other work. It have many difference as compared to state-of-the-art work ( discussed in Section 1.1 and Section 1.2). Thereafter, we highlight the uniqueness of work as follows.

- The proposed fog model is a unique architecture for mobile-cellular networks. It embeds the 5G network within fog computing. It supports the IoT applications and provides heterogeneous network connectivity. This architecture has anonymous applications in smart cities, smart homes, and the internet of everything (IoE), etc.
- The proposed model supports the device-driven communication methodology for static and dynamic mobile users. It is a very first contribution where the device-driven implemented within the fog networks where users are classified w.r.t mobility type.
- A parameter-based performance analysis with a high degree of node mobility. It helps us to check the efficiency, effectiveness, validate the work.

This paper is a bridge between the cellular network and fog computing environment. To date, fog computing is not well implemented in wireless cellular communication. The proposed work will provide an interlinking between the 5G and fog computing for better QoS/QoE. The paper provides a skeleton to design the fog network and IoE paradigm, also help to shape a scalable network management procedure within the static and dynamic network’s conditions.

## 2. Network Architecture

Fog and edge computing is an emerging area of research and its applications are added to mobile-cellular networks. It is well discussed in previous sections. Now, we discuss our core contributions. First, we talk about the proposed network architecture in Section 2.1. Therefore, we explain the advantage of the proposed schema over the traditional networks model (i.e., LTE network) and associated research challenges in Section 2.3.

### 2.1. Fog Network Architecture

**Figure 1:**
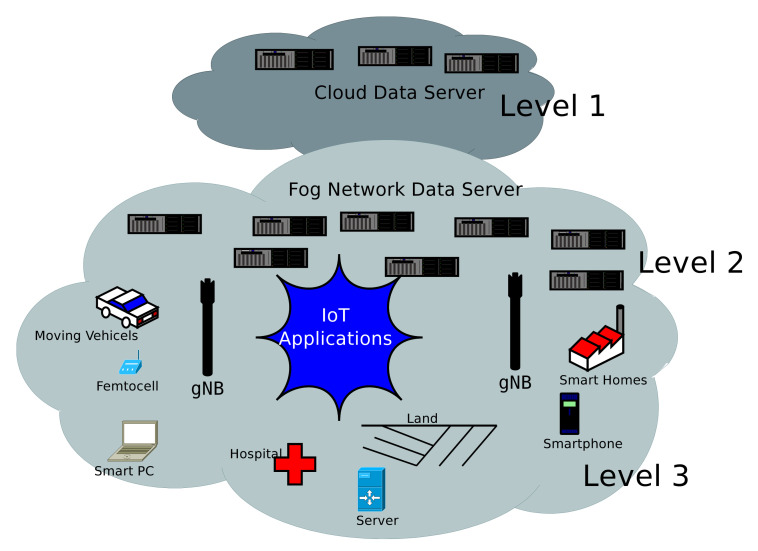
The 5G-based fog network architecture, it has three layers of independent working model to support the heterogeneous networks integration.

The proposed network architecture is the main contribution of the paper. It is based on fog networks for better QoS/QoE and mobility management to mobile nodes. The detail description is discuss in Figure 1, where a mobile node can use the device-driven communication using fog networks. This architecture support the IoT framework, green networking, energy-efficient network, and heterogeneous networks connectivity under the umbrella of 5G and fog computing.

The proposed communication network model has three levels, as shown in Figure 1. It supports computing, control, storage, acceleration, and networking for IoT applications to heterogeneous network connectivity. Level 1 contains central location-based servers and it is placed at the remote location (within the service area). Level 2 has a nearby data center and provide a fog based service environment to the end-users ( mobile devices or network enabled devices). Layer 3 is used for all connected devices to facilitate robust communication and service interface. In this paper, we consider the cloud-based IoT applications, as they are connected in the fog network, like moving vehicles, small mobile networks, local servers, smart homes, smartphones, indoor communication (hospitals and factory), etc. These peripherals are connected to the fog network at layer 3 of architecture. The computing service and network applications may demand any run-time facility to process the data or any other ongoing application. As any device starts the communication or seeking the information (services) from another user, the respective piece of information must be retrieved from the nearby fog servers to complete the requirement of the mobile node. The desired set of information must be available in mobile databases i.e., nearby fog servers.

In traditional communication or cloud-based cellular network system, the associated data is only available with central servers, where information retrieval and processing is a very challenging task. These tasks are associated with system delays and poor network performance. The proposed fog networks provide easy and reliable access to mobile data set, and it reduces the network processing complexity. As a massive number of mobile node approach to a single fog node, then fog node will serve only a limited number of end-users (until the performance is not deteriorating). Thereafter it will handover the users to nearby fog nodes. The nearby fog node will serve the user. If associated data is not available with the current fog node, then it will import the information form previously served fog nodes. The prime objective is “to maintain the performance with the possible scalable model”. The real-time deployment of fog computing in the mobile communication network is not an easy task, it has several open challenges such as latency, mobility support, scalability, resource optimization, and energy efficiency, QoS, QoE, etc. [30]. The proposed framework is a new and unique cellular network architecture to full fill mobile user expectations. It is a coupling of fog computing, cellular network system, and 5G with an IP-based backbone. It helps the mobile user to achieve a pervasive and ubiquitous communication environment over a dynamic condition including the above-listed performance parameters.

### 2.2. Working Methodology

The above-discussed model is used to define the fog network in cellular communication. Now, we elaborate on the working methodology of the proposed model using the following steps.

1. A mobile device is used to establish a connection between the target node. The call is forward toward the nearby next generation eNB (gNB), as gNB is closely associated with the mobile node.
2. The associated gNB requires mandatory data from the fog data server to serve and process the incoming call.
3. To facilitate the incoming call, the associated gNB import the mandatory information from nearby fog servers rather than back-bone data servers ( the LTE and 5G network import the information from backbone servers). In the traditional mobile networks, the required data is always fetched from central servers as they are located in a remote place.

### 2.3. Advantages

The deployment of proposed cellular network architecture has several cutting-edge advantages over the traditional telecommunication networks, as they are listed as follows.

- **User experience:** The proposed architecture provides a better network-experience to mobile users as compared to the legacy of communication technology. As a mobile user initiates the call, it go-through the several stages and processing, and finally reaches to destination side mobile node. The fog network doesn’t follow the same line of work. It always retrieves the information from fog-nodes and supports minimum infrastructure-based communication. It is a key aspect of the 5G network project. It leads us to better user experience and network performance in terms of delays and throughput.
- **Green networks:** The network-centric communication has several signal exchanges between mobile users and associated network hardware i.e., eNBs, MME, AP, and core network. It leads us toward the complexity of the networks in the system with high signaling costs. The proposed work has fewer signal overheads as compared to the legacy of networks. It has less signal exchange between the network hardware and mobile user, it concludes less signaling cost. The signaling cost is a major contribution in energy-efficient and greenness of the network.
- **Performance:** The performance of the proposed scheme is better than LTE and LTE-A networks technology. It always imports the required information from the nearby fog severs and very limited dependency on central cloud servers. Therefore, the proposed works have fast information access as compared to the network-centric schema. It leads us to high-performance network architecture.

### 2.4. Research Challenges

The proposed network architecture and it’s working is discussed in Section 2.1 and Section 2.2, respectively. The real-time deployment of the proposed system is not an easy task, it has several cutting edge research challenges, some of them are listed as follows.

- Fog nodes are densely deployed within the coverage area, the frequent database update is a mandatory task to serve the end-user devices. It is one of the critical issues in proposed network architecture.
- In fog network-based cellular system, the user’s data is available with fog servers, they are located at any place within the network service area. Therefore, security and trustworthiness is an important issue.
- Nowadays network enables devices are increasing exponentially and they are available at the end-user level. The scalability, resource allocation, programmability, and portability is another set of important research challenges. We have to address them wisely for better QoS and QoE.

## 3. Networks Communication Procedure

In this section, we present the communication methodology for the proposed work to support the dynamic and static mobile users. We consider very realistic network conditions i.e., mobile nodes are free to move in any directions, all nodes have equal capacity to receive or initiate the call. Mobile nodes follow the concept of device-centric communication as discussed in 5G specification [1]. In this paper, we extend the device-centric communication to a new level as device-driven communication within the scope of fog computing-based cellular networks.

### 3.1. Device-Driven Cellular Communication Methodology

**Figure 2:**
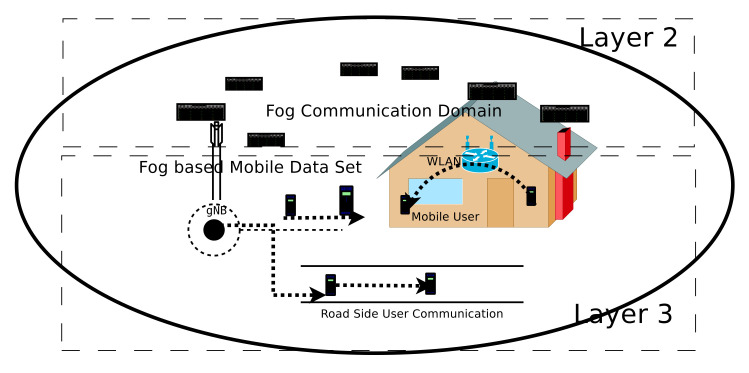
Device-driven 5G fog networks schema. It supports minimum infrastructure dependent communication between the mobile nodes and supported by fog nodes.

The proposed device-driven communication scheme will be discussed in this section. It follows the 5G-based fog networks model requirement and specification. The working methodology is elaborated in Figure 2. All associated mobile users are served by base stations (in 5G networks it’s gNB), WLAN and support the heterogeneous network connectivity. The proposed networks architecture (Section 2.1) is inspired by “natural fog“. The environmental fog is very near to the ground and we can feel them easily. The same line of thought is incorporated in cellular networks. We apply the same concept in a 5G network for better network experience. We retrieve and access the information about mobile users from the nearest fog nodes (an eNB/gNB) rather than HSS servers (as in LTE networks). The intelligent eNB and the mobile subscriber will work together to establish the device-driven communication between the source and target nodes. The 5G and beyond network have the intelligent base stations i.e, eNBs/gNBs. They are capable to manage the network load, traffic, and bandwidth consumption monitoring efficiently. The gNBs help us to maintain the mobility management where network database is extremely useful and provide guarantees for data storage, they are densely deployed in 5G and beyond network infrastructure, also called next-generation eNB (gNB).

Now, we introduce a device-driven fog network communication procedure. It has the advantages of a high data rate, less communication delay, minimum traffic towards the gNB, and capable of check the performance measurements. To elaborate it, we use Figure 2. Figure 2 have fog-based data centers, they are closed to the end-user level and all connected devices are available at this level. It facilitates the dynamic and static mobile users to improve the network’s performance and user experience. The performance metrics of edge nodes are observed by upper layer hardware as well as layer 2. The Figure 2 represents a smart cellular network, where the coverage area is equipped with gNB, WLAN, mobile database, and dynamic mobile nodes. It supports device-driven communication, where a mobile user can call another mobile node with less (or minimum) infrastructure support. In this paper, our proposed fog computing-based 5G model has two variations i.e., static user support and dynamic user support, and they are discussed in Section 3.2 and Section 3.3, respectively.

### 3.2. Network Service for Static Mobile Users

The fog computing-based 5G design provides a reliable and efficient network facility to the static mobile user.

**Figure 3:**
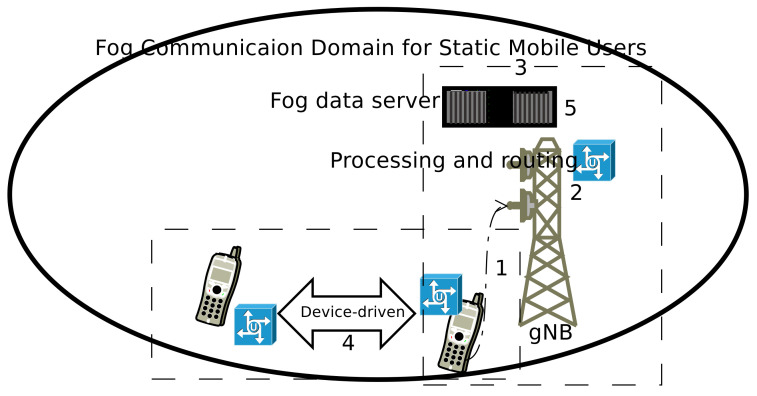
Fog network architecture for static mobile users. The nodes are using device-driven communication in the static condition.

Figure 3 show the fog network-based system for static mobile users. There are two mobile nodes (source and target) they are used to establish the connection using the following steps. The associated step is also marked in the respective figure.

1. Mobile user initiates the call, it is forward to the associated gNB (within the cell). The mobile device and associated gNB work together to find the best techniques to deliver the call towards the destination mobile node. It looks over the possibilities for, “Can they use device-driven communication to facilitate the mobile user”?
2. The mobile user and associated gNB decode the forwarded beacon signal and looking for a route to transfer the information towards the destination mobile users. The 5G and beyond cellular networks have an intelligent network identification process.
3. The mobile user and associated gNB access the desired information from the nearby fog node-based data center, because it is close to gNBs and easily accessible.
4. The gNB and mobile node find the best route for call delivery, and it is updated in the fog database. The proposed data delivery path is a single hop and gNB has a performance control over it. Here, the source and target node avail the device-driven communication methodology, therefore it is a single-hop communication. The fog node helps the mobile user’s to establish the device-driven mode of communication.
5. For other mobile users (within the same location or proximate area or similar network conditions), the information will import from fog databases (no need to process it). It will reduce the network’s computing, overheads, and delay to serve a mobile node.

### 3.3. Network Service for Dynamic Mobile Users

Now, we introduce the device-driven communication technique for dynamic mobile users. Mobile nodes are using the suggested architecture and frequently changing point of attachment.

**Figure 4:**
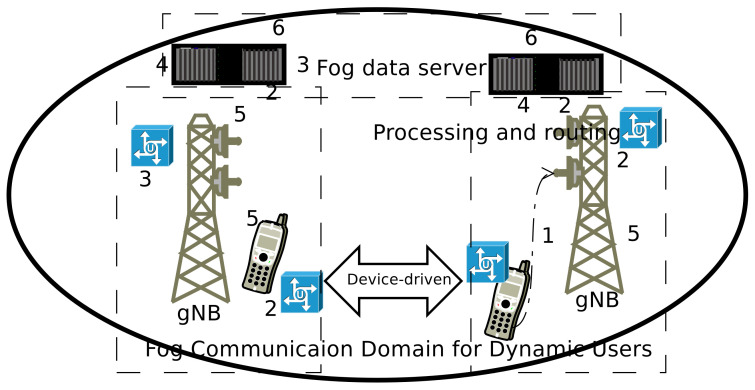
Fog network’s communication methodology for dynamic mobile users. Mobile nodes are using device-driven communication.

Figure 4 show the fog networks methodology for the dynamic node, the source, and the target node is free to move in any directions (as per the IID movement direction). In the Figure 4, there are two mobile nodes and willing to start the communication. Same time they are moving around the coverage area. The proposed communication will start with the following steps.

1. Mobile user initiates the call, it is forward to serving (associated) gNB. The device and associate gNB work together to find the best possible methodology to deliver the call towards the destination node.
  - To execute it, the main challenge is frequent node mobility. If the source node changes the position then old gNB has a lead role to facilitate the mobile user. Then old gNB flood the relevant information (about the caller and called mobile user) to all of its neighbor gNBs.
  - Thereafter new gNB serves the node (5G network follows the extreme densification of eNB/gNB, so they are deployed very close to each other). Using this facility mobile users can start device-driven communication.
2. The gNB decode the beacon signal packet and try to route the information towards the destination node. Meanwhile, If the target node changes the position, then the target gNB floods the packets to all of its neighbor gNBs.
3. The associated gNB access the desired information from the nearby available fog node databases. Then the call is delivered to the target user. The associated gNB can consider device-driven communication, subject to network performance metrics.
4. If the called mobile user is moving, then, gNB can identify the best possible method to resume the device-driven communication. To execute this condition gNB need updated data set. The generated data is frequently stored in the nearest fog data centers.
5. Now gNB, called, and caller user have the route delivery path and it should be updated on the fog data system. The data-delivery path is a single hop and gNB has a performance control over it.
6. For another mobile user (within the same location or smilier network circumstances), the information will be import form the fog networks dataset and there is no need to re-process it.

## 4. Node Mobility and Call Management Procedure

The node mobility is an important factor in the cellular network. It deals with network management and has two parts i.e., mobility management and call delivery procedure. In this section, we propose mobility management and call delivery procedures for device-driven fog networks.

### 4.1. Mobility Management Procedure

**Figure 5:**
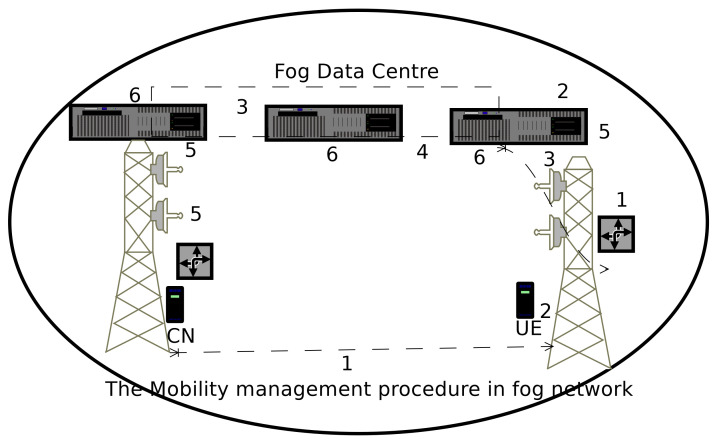
The mobility management technique in device-driven 5G fog networks.

The proposed fog network model (as discussed in Section 3.1), have a unique mobility management attribute. Its performance is dependent on node mobility and observed by mobility management. It is discussed with the following steps ( as shown in Figure 5).

1. The mobile node changes the location from one gNB to another nearby gNB, and the user initiates a call. Therefore, a new mobility management procedure is required for better QoS and QoE.
2. The mobile user seeks a new point of attachment for network services. The associated gNB and mobile users work together to find a nearby point of attachment (from where users can get network information and services?).
3. To serve a mobile node, network, mobile user and gNB seek the mandatory information for location update and location registration procedure. Same time the mobile user and gNB exchange a few signals to establish a connection between the mobile user and gNB.
4. The mobile node and associated gNB import the information about the called and caller user from the nearest fog data centers.
5. As the called and caller mobile user changes the point of attachment, the associated gNB flood the information to its neighboring gNBs. Thus they do not re-import the information from the databases, just utilize it. Also, put an effort to find the minimum metric-based techniques to maintain the QoS and QoE parameters.
6. The modified information to be stored in the nearest fog data center, it helps us to precise the current location of the mobile users. If the source and target node change the position, the gNB help to establish the route between them. The source node puts effort for self reconfigurability and packet delivery, it also maintains the performance metrics i.e., throughput and delay.

### 4.2. Call Delivery Procedure

**Figure 6:**
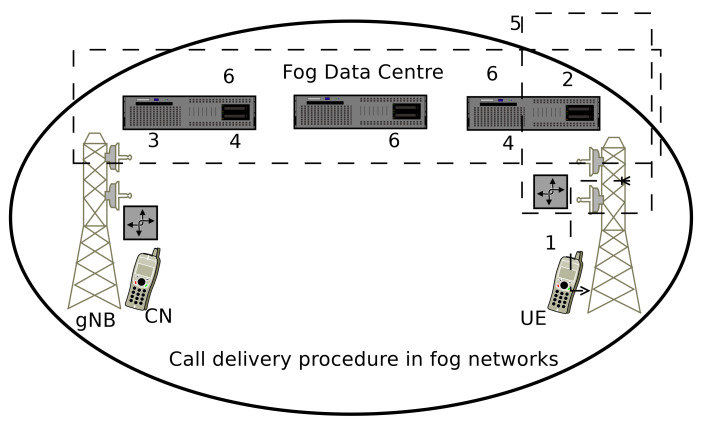
Call delivery procedure in fog computing-based device-driven 5G networks.

As the mobility management procedure is completed, thereafter the call should be delivered to the destination node. The call delivery is an important role in mobility management as discussed in Section 4, and it has the following steps (discuss with Figure 6).

1. The mobile user starts the call, and it is forwarded to associated nearby gNB.
2. The associated gNB and mobile node fetch the required information from the nearest fog data centers.
3. The current gNB forwards the call and required information towards the target gNB.
4. The target gNB delivers the call to the mobile user.
5. If the called mobile user changes the point of attachment, then associated gNB flood the information to neighboring gNBs.
6. The respective information must be updated in fog data centers for future references. This process reduces the signaling cost to the network. Overall it improves the energy efficiency and greenness of the networks.

## 5. Advantages and Cutting Edge Research Challenges

In Section 2, Section 3 and Section 4, we have discussed the fog network-based architecture, device-driven communication methodology and unique mobility management procedure. Now we discuss the advantage and associated research challenges of them.

### 5.1. Advantages

The suggested communication methodology is a new paradigm for fog computing, where using cellular networks. It has the following advantages over traditional cellular network technology.

1. **Scalability and robustness:** The exponential growth of networks associated devices and the majority of them have IoT enabled interfaces, and it generates a sheer amount of network data. The fog network architecture provides the scalability to network services because it follows the local device-centric design rather than the central server topology.
2. **Mobility support:** Mobility management is an important aspect of fog networks and communication. It is more focused because the nodes are using device-driven communication. We are aware of the fact of sophisticated data access techniques from the central server and capabilities, as it is a very complex task in LTE/LTE-A networks. The fog-based 5G network provides dynamic mobility management, it allows a small number of gateway devices to manage a large number of edge devices (IoT connected devices) [31].
3. **Least delay:** The fog networks provide instant and smooth access to desired data form nearby fog data centers. As the mobile node changes the position or call another mobile user, the required information (for the call delivery and mobility management) will retrieve from the nearest fog data center, and it requires less call setup time as compared to seek from the central cloud servers (used by LTE/LTE-A networks).
4. **Storage management:** The storage management encompasses with associated technologies and data management processes. The organizations work together on these factors to maximize or improve the performance of the system as well as efficient data storage resources. The proposed fog network retrieves the information from the nearest fog server rather than the central cloud servers. Here, the data are distributively stored and easily accessible, and avoiding the deadlock and queuing procedure.
5. **Load balancing:** The load balancing is an effective approach to address the data rate fluctuation problem for 5G cellular networks [32]. The channel borrowing from neighboring cells is not well applicable in 5G wireless networks. Then the fog network is a possible solution for it. Where, the network computing, service, user credential, and performance data are taken from the fog data center. Here device-driven communication is advocated to facilitate load balancing without an extra load on the data servers. It will provide the spectrum efficiency, less data traffic, offloaded the congested cell, etc.
6. **Traffic management:** The 5G network is the possible solution for an increasing number of mobile users. It has various real-time deployment areas such as vehicle-to-vehicle communication and efficient traffic management, it is a serious issue in metropolitan cities. A traffic management based four-tier network architecture is proposed in [24], with the convergence of VANETs, 5G networks, software-defined networks, and mobile edge computing technologies. The real-time deployment of this network and the fog network will be an efficient contribution to fog networks.

### 5.2. Open Research Challenges

The proposed network architecture has following open research challenges for academia and industry.

1. **Security:** The fog network is an emerging paradigm where mobile users can use on-demand network services with performance metrics. These services can be achieved by dense deployment of the fog data center for information retrieval. It leads to data duplication, also can hamper the security and privacy of individual persons.
2. **Energy efficiency:** To implement the fog network, energy consumption is a major challenge, because it has several signal exchange. It leads us to high energy consumption in communication. This energy requirement is less than the current network-centric communication system. In fog network, most of the signal exchange is limited to end-users and fog nodes. Our main objective to reduce it to an optimal level. The authors of Ref. [33] proposed a modern fog network architectures and mobility management scheme with IoT applications in 5G communications technologies. It consists of a huge number of mobile nodes and frequent node mobility and handovers. The proposed work used the blockchain for QoE in networks.
3. **Data management and routing:** The fog based network is an easy way to achieve the high data rate, but it also generates a “Big Data“ because of multiple data-processing modes. As the growing data should be delivered at the destination fog data server/node, the routing is another groundbreaking challenge. The authors of Ref. [34] discussed the associated matrices for data-centric fog network, input data size characteristics, and data flow properties.

## 6. Performance Analysis and Result Discussion

In this section, we discuss the performance analysis and comparison of proposed work. To implement it, we introduce some notations as they are listed in Table 1.

The efficiency and effectiveness of the proposed work are being checked by a simulation setup and numerical model. Let, there are *n* number of mobile users and *N* number of gNBs within the service area *A*, associated fog data centers are *M*. The fog data centres are deploy in a homogeneous environment with a set of $S_{i}=1,\;2,\;3,\;\ldots ,\;|S|$. The fog nodes are used to share the computation of mobile resources with end-users, as shown in Figure 1. In the proposed network model, each fog data centers are assisted by nearby fog network controller or independent fog devices. These nodes are capable to execute the intensive tasks such as routing and performance observation etc. The fog data centers also used to import and export information from nearby fog nodes. They are densely deployed with nearby gNBs. Thus, it can be accessed by the gNB or independently by fog data centers. In other words, the signal to interference noise ratio (SINR) should be higher than a threshold value ($\Psi_{min}$) to deliver the correct/complete data. We define the received SINR from a mobile user ($u_{i}$) to fog node at position ($fn_{i}^{j}$) using the link ($w_{k}^{j}$) as follows, the i,j,k are the points within a service area.

(1) $$\Psi =\frac{P_{i}g_{i,j}^{k,l}}{\sum_{u=1}^{n}r_{i,j}^{k,l}h_{i,j}^{k,l}+\sigma_{N}^{2}},$$

In Equation (1), $P_{i}$ and $g_{i,j}^{k,l}$ is the transmission power and channel gain between the mobile user $u_{i}$ and fog node. The $fn_{k,l},h_{i,j}^{k,l}$ represents the interference channel gain from other mobile node and $\sigma_{N}^{2}$ is the channel noise. If it satisfies SINR requirement then it must follow the following condition of Equation (2),

(2) $$S_{R}=w_{k,l}^{i,j}log\left(1+\Psi \right).$$

In Equation (2),

(3) $$w_{i,j}^{k,l}=\frac{P_{i}g_{i,j}^{k,l}}{\sum_{u=1}^{n}P_{j}h_{i,j}^{k,l}}.$$

We assume the call setup time is *T*, and it depends on the time taken to establish a call between the source and target mobile nodes. For better QoE, the call setup delay is an important parameter. The most considerable point is that it should guarantee the transmission quality between the users and the fog data center, as per the expectation of 5G networks. Then the total call setup delay is,

(4) $$T_{cst}=\sum_{0}^{\delta t}\left(3t_{prop}+3t_{proc}+2t_{trasn}\right).n,D_{p}.MC.$$

In Equation (4) MC is mobility coefficient and we can write it as follows in Equation (5),

(5) $$MC=\xi \sum_{i}^{H}p_{i}\int_{0}^{d}tdt.$$

In Equation (4) $t_{prop},t_{proc},t_{trasn}$ represent the propagation time, processing time and transmission time for the channel. The *n* is the number of mobile users, and $D_{p}$ is the size of the data packet. ξ is hop counts, $H,p_{i}$ the total movement directions (H = 6) and probability, *d* is the distance thresholds. $T_{cst}$ is total call setup time. δt is call duration. In this work, we consider initial simulation parameters as follows. There are 1000 and 150 mobile users and fog data centers, respectively. Active gNBs are 10 and a cell (5G follows the extrema deification of a base station). The minimum hop counts for reliable communication is 2, user movement directions are 6 (as per the IID), initial movement probability 0.01, call duration is 120 s, distance from the last location is 20 m, and the call arrival service rate $\lambda_{i}=0.01$, the call departure service rate is $\mu_{i}=0.001$. The network antenna gain for transmission and receiver is 45 dBm and 43 dBm, respectively. The active eNB is 20, and at least 10 active users per cell, and cell-radius is 50 m, user-mobility-distance threshold is 5 cells crossing, the average interference margin is 5.5 dB.

The call-to-mobility (CMR) is an important point to conclude the network performance. It provides the actual and real-time network performance where mobile nodes are moving as well as seeking network services. In this paper, we calculate it as follows,

(6) $$CMR=\frac{\lambda_{i}}{\mu_{i}}.$$

In Equation (6) λ and μ are the service rate for call arrival and departure.

**Figure 7:**
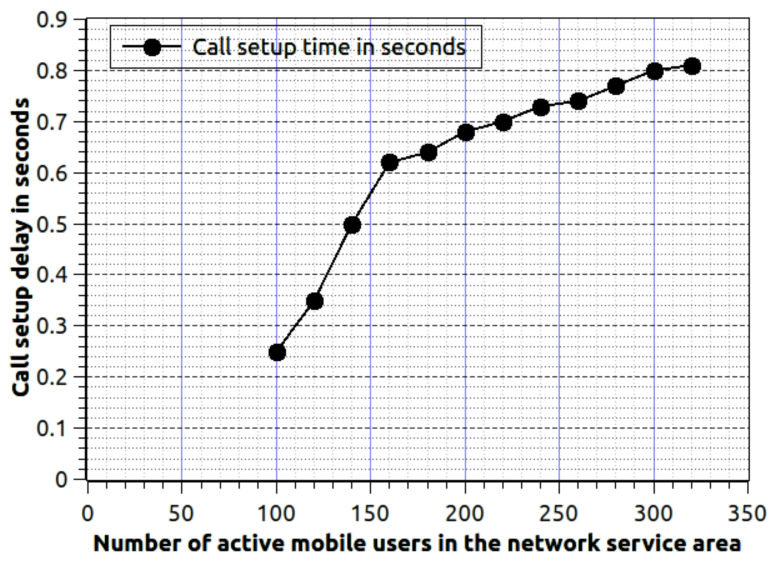
Number of active mobile users in the network service area verses call setup delay, where $\lambda_{i}=0.01$, $\mu_{i}=0.001$ and δt=120 s.

Figure 7 show the efficiency of proposed work, while the number of mobile user increases and we are concerning about the call setup delay. We estimate the call setup delay is the total time (in seconds) require to deliver the first packet to the target mobile node. The number of mobile users varies from 100 to 320, the call arrival rate $\lambda_{i}=0.01$, and departure rate is $\mu_{i}=0.001$. As the number of mobile users are growing then the associated delay must be increased. If the numbers of active users between are 100 to 150, then the delay increase in a higher rate. Because the network sends the information exchange signal to nearby fog nodes to serve the mobile nodes. Once the data is available to all fog nodes, then the delay acceleration rate becomes slow. This effect is visible in Figure 7. If the number of mobile nodes is very high, for example, 500 nodes then the delay increment rate will be the same. It is completely subject to fog nodes signal exchange mechanism.

**Figure 8:**
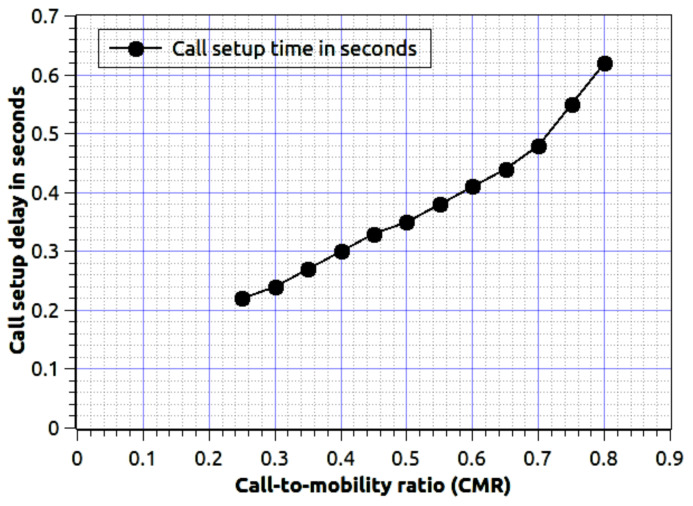
Call-to-mobility ratio verses call setup delay. We consider $\lambda_{i}$ and $\mu_{i}$ = 0.001, n = 100, M = 1000 and node velocity = 50 km/h.

Figure 8 represents the CMR ratio verses the call setup delay for proposed fog-based network schema. The low CMR refers to low mobility of nodes and high call arrival, and high CMR is vies-versa. The proposed work has less call setup delay as low node mobility because the required data is quickly available to establish a network connection. As the mobility is high the mobile nodes exchange several signals to all nearby fog data centers, thus it leads us to high call setup delay. To simulate CMR we consider $\lambda_{i}$ and $\mu_{i}$ = 0.001, n = 100, M = 1000 and node velocity = 50 km/h. If the number of mobile nodes is very high, i.e., 500 users, then the associated delay is dependent on CMR value. Assume we consider the low CMR, then delay is will not grow rapidly. For the high value of CMR, the delay will increase exponentially, because the nodes are very dynamic and they are using network services. To serve a mobile node, the fog nodes require the latest data set, and it affects the delay.

The performance of the proposed work depends on the number of fog nodes deployed in the service area. Active fog nodes have a major role in system performance.

**Figure 9:**
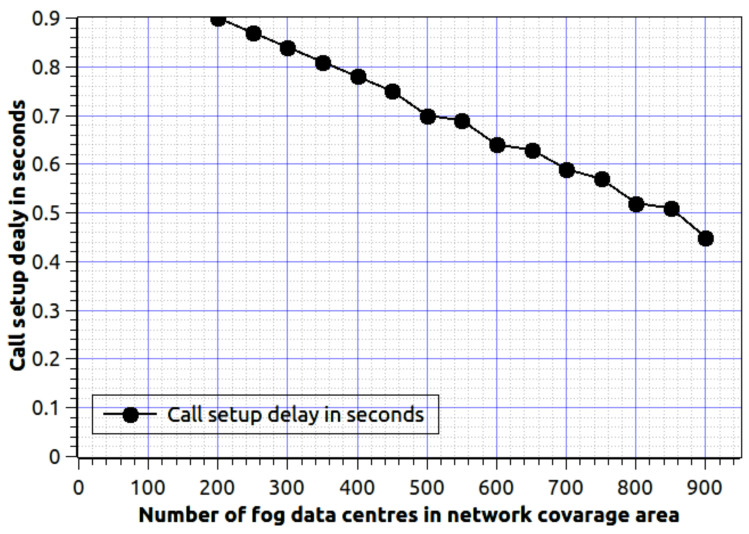
Number of the fog data center in the network service area verses call setup delay.

Figure 9 shows the effect of fog data center over the call setup delay. Here, the λ=0.01 and $\mu_{i}=0.001$ average number of mobile users are 1000 in each fog data centre. As the number of fog servers are increases the call setup delay is reducing. Because, fog nodes have the information to serve the mobile nodes. Therefore, no need to retrieve and process the new data. It will reduce the call set-up delay.

**Figure 10:**
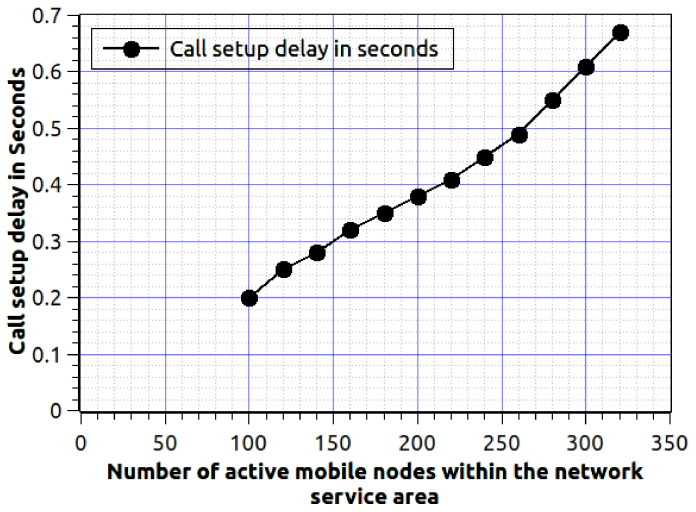
Number of mobile users within the network service area verses CMR.

Figure 10 shows the effect of active mobile user versus calls setup delay, here we consider CMR also. It will help us to understand the system’s robustness, and proved through the graph. Proposed work provides worthy scalability and robustness for mobile users. The parameters are as follows, λ=0.01, μ=0.01, n = 1000, N = 200, M = 500 and CMR = 0.4. In the figure, we can observe that, as the number of active mobile nodes is growing the call setup delay is accelerating. Here, we consider 1000 mobile users (within the network coverage area), out of the 320 users ( at max) are active (availing the network services, non-ideal mode).

**Figure 11:**
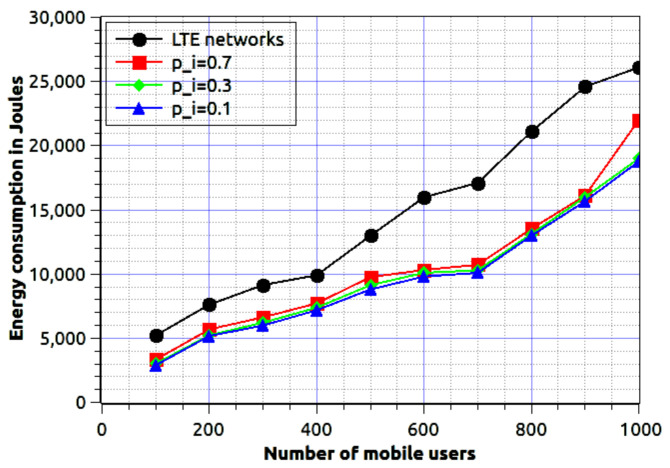
Number of active mobile users using device-driven communication with various level of node mobility verses energy consumption in Joules.

In this paper, we compare our results with LTE networks. It will help us to check the superiority of the proposed work. We consider the LTE network is one of the most successful and deployed cellular networks, therefore we map the proposed work with it. Figure 11 shows energy consumption for fog network and LTE networks. We consider the various level of node mobility i.e., 0.1, 0.3, and 0.7. Other simulation parameters are as follows, the number of mobile users varies from 100 to 1000, active gNBs are 200, fog nodes are 300. For the LTE network, we consider 0.4 as the node mobility because it is a fair value of movement probability. If it is higher than 0.6, then the user is more dynamic, if less than 0.4 then almost a static user. Thus, we consider 0.4 as movement probability. Obtained results are better in the proposed work because it does not import the information’s from central servers. It always tries to import the data from fog nodes and establish device-driven communication. In our next work, we will compare the results w.r.t fog networks based communication as suggested in [25,26].

## 7. Conclusions

Fog network is the advancement in the current state-of-the-art for modern cellular networks. In this paper, we proposed a new design and framework for fog-based 5G networks. It has a reliable and fast communication procedure with high node mobility. The suggested networks model is a future networks-schema for high demanding greedy mobile users. The mobile nodes access and store desired pieces of information from the nearby fog data center, and established a device-driven communication system between the source and target mobile users, and it uses limited network dependency during communication. The proposed work provides better network output in terms of delay, latency, and data rates. In this paper, we analyze the performance of the system in terms of call setup delay, we can explore the performance parameters for mobility support, security, scalability, storage management, and load balancing, etc. Thus, we can conclude the fog networks is the future of next-generation cellular network system.

## Figures and Tables

**Table 1:** Notation and respective descriptions.

Notation	Description
gNB	Next generations evolve Node Base station.
δt/t	Call duration/total time.
$t_{proc}$	Processing time.
$t_{trans}$	Transmission time.
$t_{prop}$	Propagation time.
n/N	Number of mobile nodes/gNB.
$D_{p}$	Size of the data packets.
ξ	Number of hop counts.
$H,p_{i}$	Movement directions, and probability.
*d*	Distance thresholds.
$T_{cst}$	Total call setup delay.
UE/CN	User equipment/Corresponding node.
A/M	Service area/Number of the fog data center.
$\Psi_{min}$	Minimum threshold value of the quality parameter.
$P_{i}$	Transmission power.
$g_{i,j}^{k,l}$	Channel gain between user *i* and fog node.
$fn_{k,l}$	Interference channel gain from other mobile node.
$\sigma_{N}^{2}$	Channel noise.
MC	Mobility coefficient.
CMR	Call to mobility ratio.
λ/μ	Call arrival/departure rates.

## References

[1] Andrews J.G., Buzzi S., Choi W., Hanly S.V., Lozano A., Soong A.C.K., Zhang J.C. (2014). What Will 5G Be?. IEEE J. Sel. Areas Commun..

[2] Karlsson A., Al-Saadeh O., Gusarov A., Challa R.V.R., Tombaz S., Sung K.W. Energy-efficient 5G deployment in rural areas. Proceedings of the 2016 IEEE 12th International Conference on Wireless and Mobile Computing, Networking and Communications (WiMob).

[3] Babu S., Biswash S.K. (2019). Fog computing based node-to-node communication and mobility management technique for 5G networks. Trans. Emerg. Telecommun. Technol..

[4] Navarro E., Costa N., Pereira A. (2020). A Systematic Review of IoT Solutions for Smart Farming. Sensors.

[5] Chaudhary R., Kumar N., Zeadally S. (2017). Network Service Chaining in Fog and Cloud Computing for the 5G Environment: Data Management and Security Challenges. IEEE Commun. Mag..

[6] Jiang Y. (2019). Cooperative caching in fog radio access networks: A graph-based approach. IET Commun..

[7] Mukherjee M., Shu L., Wang D. (2018). Survey of Fog Computing: Fundamental, Network Applications, and Research Challenges. IEEE Commun. Surv. Tutor..

[8] Mouradian C., Naboulsi D., Yangui S., Glitho R.H., Morrow M.J., Polakos P.A. (2018). A Comprehensive Survey on Fog Computing: State-of-the-Art and Research Challenges. IEEE Commun. Surv. Tutor..

[9] Ali M.S., Hossain E., Kim D.I. (2017). LTE/LTE-A Random Access for Massive Machine-Type Communications in Smart Cities. IEEE Commun. Mag..

[10] Biswash S.K., Jayakody D.N.K. (2018). Performance based user-centric dynamic mode switching and mobility management scheme for 5G networks. J. Netw. Comput. Appl..

[11] Tayyab M., Gelabert X., Jäntti R. (2019). A Survey on Handover Management: From LTE to NR. IEEE Access.

[12] Biswash S.K., Jayakody D.N.K. (2020). Energy-efficient node-to-node communication scheme for fog-based cellular networks. IET Commun..

[13] Riekstin A.C., Nguyen K.K., Rodrigues B.B., de Brito Carvalho T.C.M., Meirosu C., Stiller B., Cheriet M. (2018). A Survey on Metrics and Measurement Tools for Sustainable Distributed Cloud Networks. IEEE Commun. Surv. Tutor..

[14] Yang S. (2017). IoT Stream Processing and Analytics in the Fog. IEEE Commun. Mag..

[15] Trakadas P., Nomikos N., Michailidis E.T., Zahariadis T., Facca F.M., Breitgand D., Rizou S., Masip X., Gkonis P. (2019). Hybrid Clouds for Data-Intensive, 5G-Enabled IoT Applications: An Overview, Key Issues and Relevant Architecture. Sensors.

[16] Shiraz M., Gani A., Khokhar R.H., Buyya R. (2013). A Review on Distributed Application Processing Frameworks in Smart Mobile Devices for Mobile Cloud Computing. IEEE Commun. Surv. Tutor..

[17] Sanaei Z., Abolfazli S., Gani A., Buyya R. (2014). Heterogeneity in Mobile Cloud Computing: Taxonomy and Open Challenges. IEEE Commun. Surv. Tutor..

[18] Perera C., Qin Y., Estrella J.C., Reiff-Marganiec S., Vasilakos A.V. (2017). Fog Computing for Sustainable Smart Cities: A Survey. ACM Comput. Surv..

[19] Aazam M., Zeadally S., Harras K.A. (2018). Fog Computing Architecture, Evaluation, and Future Research Directions. IEEE Commun. Mag..

[20] Sookhak M., Yu F.R., He Y., Talebian H., Safa N.S., Zhao N., Khan M.K., Kumar N. (2017). Fog Vehicular Computing: Augmentation of Fog Computing Using Vehicular Cloud Computing. IEEE Veh. Technol. Mag..

[21] Virdis A., Vallati C., Nardini G., Tanganelli G., Stea G., Mingozzi E. (2018). D2D Communications for Large-Scale Fog Platforms: Enabling Direct M2M Interactions. IEEE Veh. Technol. Mag..

[22] Ren H., Pan C., Liu N., Yu X.H., Elkashlan M., Nallanathan A., Hanzo L. (2018). Low-Latency C-RAN: A Next-Generation Wireless Approach. IEEE Veh. Technol. Mag..

[23] Kitanov S., Janevski T. State of the art: Fog computing for 5G networks. Proceedings of the 24th Telecommunications Forum (TELFOR).

[24] Liu J., Wan J., Jia D., Zeng B., Li D., Hsu C.H., Chen H. (2017). High-Efficiency Urban Traffic Management in Context-Aware Computing and 5G Communication. IEEE Commun. Mag..

[25] Aggarwal S., Kumar N. (2019). Fog Computing for 5G-Enabled Tactile Internet: Research Issues, Challenges, and Future Research Directions. Mob. Netw. Appl..

[26] Khalid O., Khan I.A., Rais R.N.B., Malik A.W. (2020). An Insight into 5G Networks with Fog Computing. Fog Computing.

[27] Choi N., Kim D., Lee S.J., Yi Y. (2017). A Fog Operating System for User-Oriented IoT Services: Challenges and Research Directions. IEEE Commun. Mag..

[28] Oteafy S.M.A., Hassanein H.S. (2018). IoT in the Fog: A Roadmap for Data-Centric IoT Development. IEEE Commun. Mag..

[29] Darwish T.S.J., Bakar K.A. (2018). Fog Based Intelligent Transportation Big Data Analytics in The Internet of Vehicles Environment: Motivations, Architecture, Challenges, and Critical Issues. IEEE Access.

[30] Zhang W., Zhang Z., Chao H.C. (2017). Cooperative Fog Computing for Dealing with Big Data in the Internet of Vehicles: Architecture and Hierarchical Resource Management. IEEE Commun. Mag..

[31] Ni J., Zhang A., Lin X., Shen X.S. (2017). Security, Privacy, and Fairness in Fog-Based Vehicular Crowdsensing. IEEE Commun. Mag..

[32] Zhang H., Song L., Zhang Y.J. (2018). Load Balancing for 5G Ultra-Dense Networks using Device-to-Device Communications. IEEE Trans. Wirel. Commun..

[33] Sharma V., You I., Palmieri F., Jayakody D.N.K., Li J. (2018). Secure and Energy-Efficient Handover in Fog Networks Using Blockchain-Based DMM. IEEE Commun. Mag..

[34] Mahmud R., Kotagiri R., Buyya R., Di Martino B., Li K.C., Yang L.T., Esposito A. (2018). Fog Computing: A Taxonomy, Survey and Future Directions. Internet of Everything: Algorithms, Methodologies, Technologies and Perspectives.

